# Tracked Foley catheter for motion compensation during fusion image-guided prostate procedures: a phantom study

**DOI:** 10.1186/s41747-020-00147-4

**Published:** 2020-04-16

**Authors:** Graham R. Hale, Filippo Pesapane, Sheng Xu, Ivane Bakhutashvili, Neil Glossop, Baris Turkbey, Peter A. Pinto, Bradford J. Wood

**Affiliations:** 1 Center for Interventional Oncology, Radiology and Imaging Sciences, National Cancer Institute, National Institutes of Health, Bethesda, MD USA; 2 Urologic Oncology Branch, National Cancer Institute, National Institutes of Health, Bethesda, MD USA; 3 Postgraduate School in Radiodiagnostics, Università degli Studi di Milano, Milan, Italy; 4 Arcitrax, Toronto, Canada; 5 Molecular Imaging Program, National Cancer Institute, National Institutes of Health, Bethesda, MD USA

**Keywords:** Focal therapy, Prostatic neoplasms, Image-guided biopsy, Spatial navigation, Surgery (computer-assisted)

## Abstract

**Background:**

Uncorrected patient or prostate motion may impair targeting prostate areas during fusion image-guided procedures. We evaluated if a prototype “tracked Foley catheter” (TFC) could maintain fusion image alignment after simulated organ motion.

**Methods:**

A pelvic phantom model underwent magnetic resonance imaging (MRI), and the prostate was segmented. The TFC was placed in the phantom. MRI/ultrasound (US) fusion was performed. Four trials were performed varying motion and TFC presence/absence: (1) TFC/no-motion, (2) TFC/motion, (3) no-TFC/no-motion, and (4) no-TFC/motion. To quantify image alignment, screen captures generated Dice similarity coefficient (DSC) and offset distances (ODs) (maximal US-to-MRI distance between edges on fusion images). Three anatomical targets were identified for placement of a needle under fusion guidance. A computed tomography scan was used to measure system error (SE), *i.e.,* the distance from needle tip to intended target.

**Results:**

The TFC presence improved MRI/US alignment by DSC 0.88, 0.88, 0.74, and 0.61 in trials 1, 2, 3, and 4, respectively. Both OD (trial 2 *versus* trial 4, 4.85 ± 1.60 *versus* 25.29 ± 6.50 mm, *p* < 0.001) and SE (trial 2 *versus* trial 4, 6.35 ± 1.31 *versus* 32.16 ± 6.50 mm, *p* < 0.005) were significantly lower when the TFC was present after artificial motion, and significantly smaller OD when static (trial 1 *versus* trial 3, 4.29 ± 1.24 *versus* 6.42 ± 2.29 mm, *p* < 0.001).

**Conclusion:**

TFC provided better image alignment with or without simulated motion. This may overcome system limitations, allowing for more accurate fusion image alignment during fusion-guided biopsy, ablation, or robotic prostatectomy.

## Key points

- Outcomes for image-guided prostate biopsy/ablation are dependent upon accurate needle placement.
- Multimodality image fusion allows intra-procedural localisation of tool position and orientation.
- A custom tracked Foley catheter maintained appropriate magnetic resonance imaging/ultrasound-fusion image alignment after movement.
- Tracked Foley catheter may correct intra-procedural prostate motion during fusion-guided prostate procedures.

## Background

Within the last decade, fusion image-guided procedures have been applied for the diagnosis and treatment of prostate cancer (PCa). PCa is the most common non-cutaneous cancer in American men: it has been estimated that 164,690 new cases will be diagnosed and that 29,430 American men will die of PCa in 2018 [1].

Recent urologic PCa guidelines have suggested the use of multiparametric MRI (mpMRI) in biopsy naïve men or those with prior negative prostate biopsy [2, 3]. The mpMRI/ultrasound (US) fusion-guided prostate biopsy (FBx) platforms address some of the major limitations of transrectal ultrasound (TRUS)-guided prostate biopsy [4, 5]. Combining the unique capacity to detect clinically significant PCa with the real-time versatility of US [6, 7], FBx increases the accuracy of PCa localisation compared to conventional (blind and random) TRUS biopsies [8, 9], demonstrating higher rates of diagnosis of clinically significant PCa being diagnosed, while simultaneously not detecting indolent PCa that may not require treatment [5, 9, 10]. FBx highlights the benefits that fusion-guided procedures may offer patients; however, challenges and limitations remain. Currently, outcomes for image-guided prostate biopsy and focal ablation are highly dependent upon accurate needle placement at an exact target location [7, 8, 10–14]. Semi-automated computer navigation and guidance allow for the registration (alignment/matching) of pre-procedural imaging (*i.e,* mpMRI) with real-time intra-procedural imaging (*i.e,* US). This multimodality image fusion is dependent upon accurate registration of multiple image datasets (mpMRI and US), allowing intra-procedural localisation of the exact position and orientation of tools [15]. Together, registration and fusion enable pre-identified locations to be targeted for biopsy or ablation [16]. After initial MRI/US image registration and fusion, the US transducer is spatially tracked to provide intra-procedural guidance [7]. However, performance of current FBx platforms and software may markedly degrade during the procedure, due to uncorrected patient or organ motion, or shape deformations. Manual correction of prostate motion and shape deformation can be unreliable due to poor signal-to-noise ratio, out-of-plane prostate motion, and the inability to correct two-dimensional correlations, much less three-dimensional correlations in other planes [16, 17]. Moreover, manual rigid or elastic adjustment of registration may cause offset errors. Likewise, fusion image misalignment may result from intra-procedural repeat registration after organ shift or motion.

**Fig. 1:**
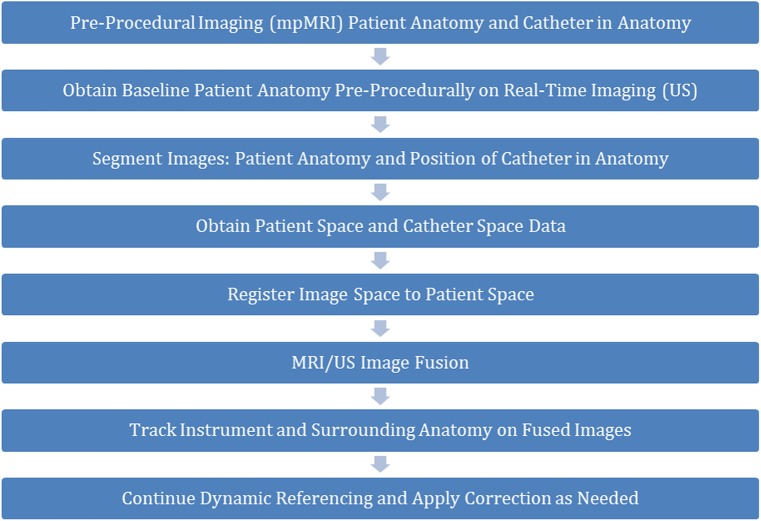
Workflow incorporating the tracked Foley catheter

**Fig. 2:**
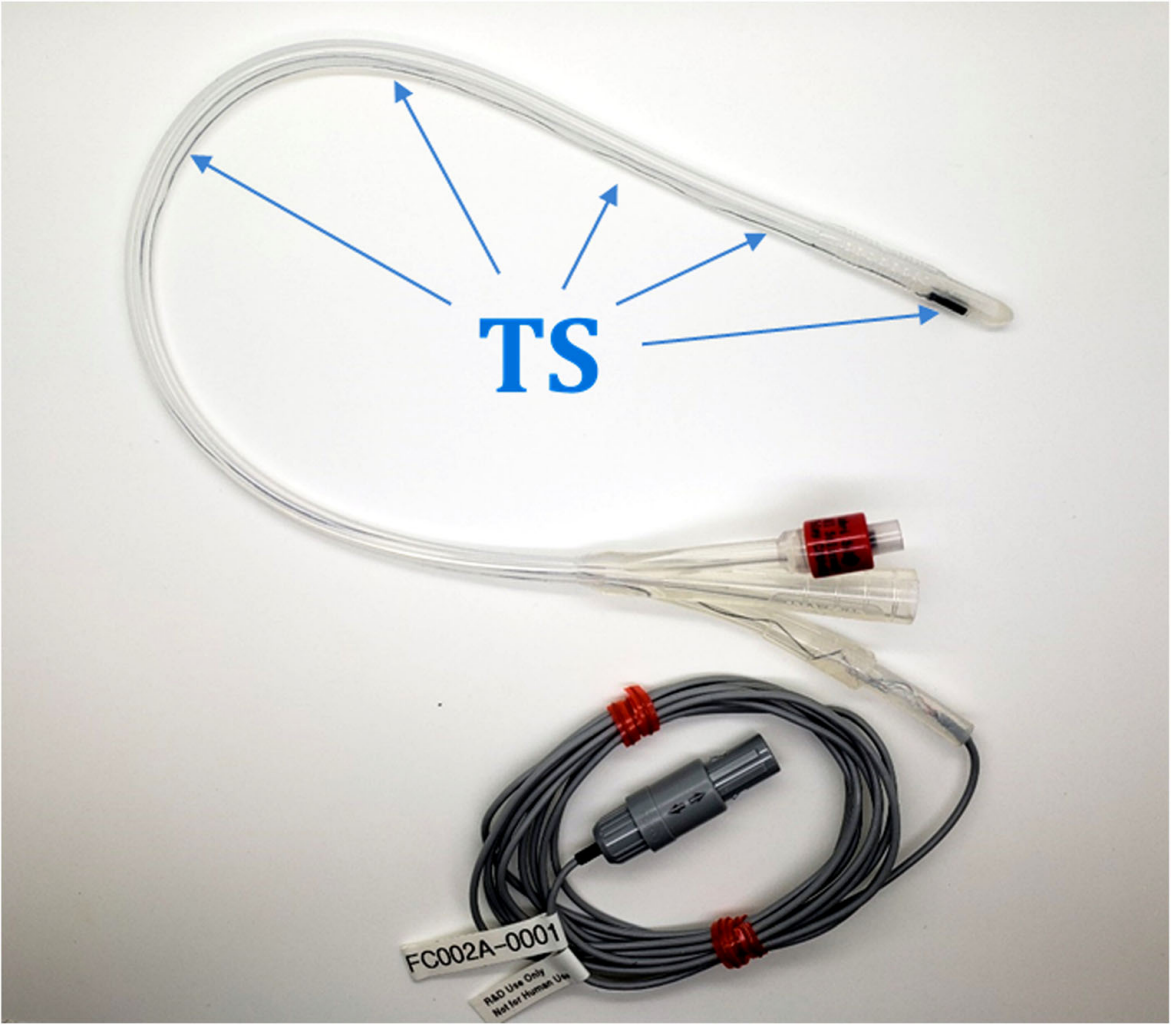
Tracked Foley catheter (TFC) prototype. The TFC has a six degrees of freedom (forward/backward, up/down, left/right, and rotational movement along *x*, *y*, and *z* axes) tracking sensor (TS) that assumes a complex shape in the urethra and provides real-time tracking data

A modified Foley catheter with embedded sensors may help correcting for fusion image mismatch, but requires adjustment of the fusion system, methods, and instrumentation (Fig. 1) [18]. With direct real-time feedback of prostate motion (dynamic referencing), this custom tracked Foley catheter (TFC) may provide motion compensation to address the problem of MRI/US misalignment during procedures. This solution uses the same electromagnetic tracking principles as FBx platforms (Fig. 2), and it may improve current system limitations allowing for more accurate image alignment during fusion procedures, such as biopsy, focal PCa ablation, or even possibly fusion-guided robotic prostatectomy in the future, although speculative [15, 19–24]. The present phantom study aimed to compare fusion image alignment after simulated prostate motion with and without the correction provided by the TFC.

## Methods

This study did not involve human subjects or patient data and thus did not require institutional review board approval. Two TFC FBx inexperienced users (one urologist/staff scientist, one research fellow) and one expert fusion software computer science engineer participated in study design and data collection.

**Fig. 3:**
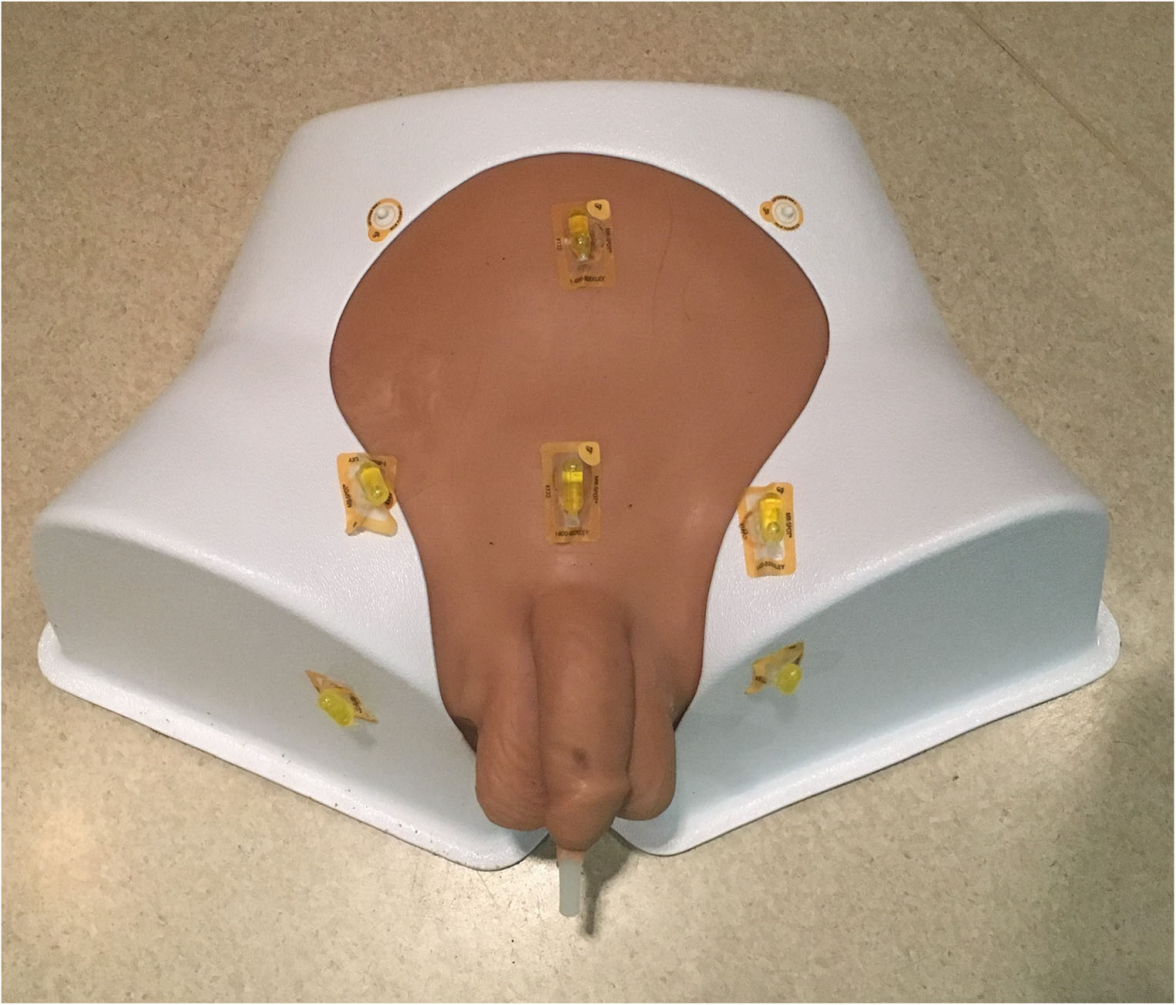
Custom phantom model. This experiment was performed on a custom pelvic anthropomorphic phantom model. The model contains a catheterisable urethra and urinary bladder, prostate, and surrounding prostatic structures oriented anatomically and visualised on magnetic resonance imaging (MRI), computed tomography (CT), and ultrasound. CT and MRI fiducial markers, which measured simulated motion, are visible on the model surface

**Fig. 4:**
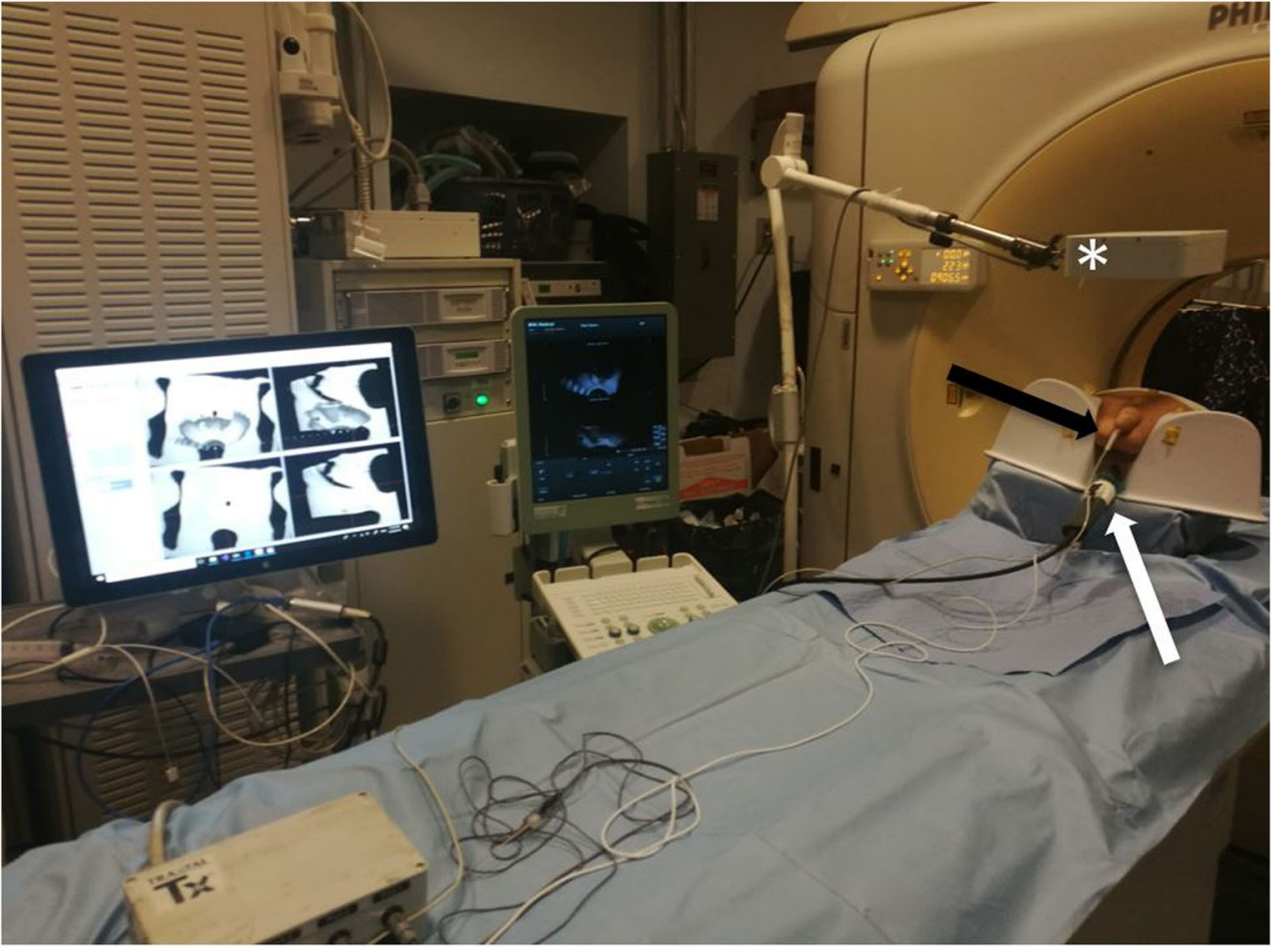
Tracked Foley catheter (TFC) test-bed experiment. The screen on the left shows the custom fusion biopsy software platform (Onconav, National Institutes of Health, USA). The TFC prototype (*black arrow*) enters the anthropomorphic phantom urethra. Ultrasound transducer in phantom rectum (*white arrow*). Electromagnetic field generator (*asterisk*)

**Fig. 5:**
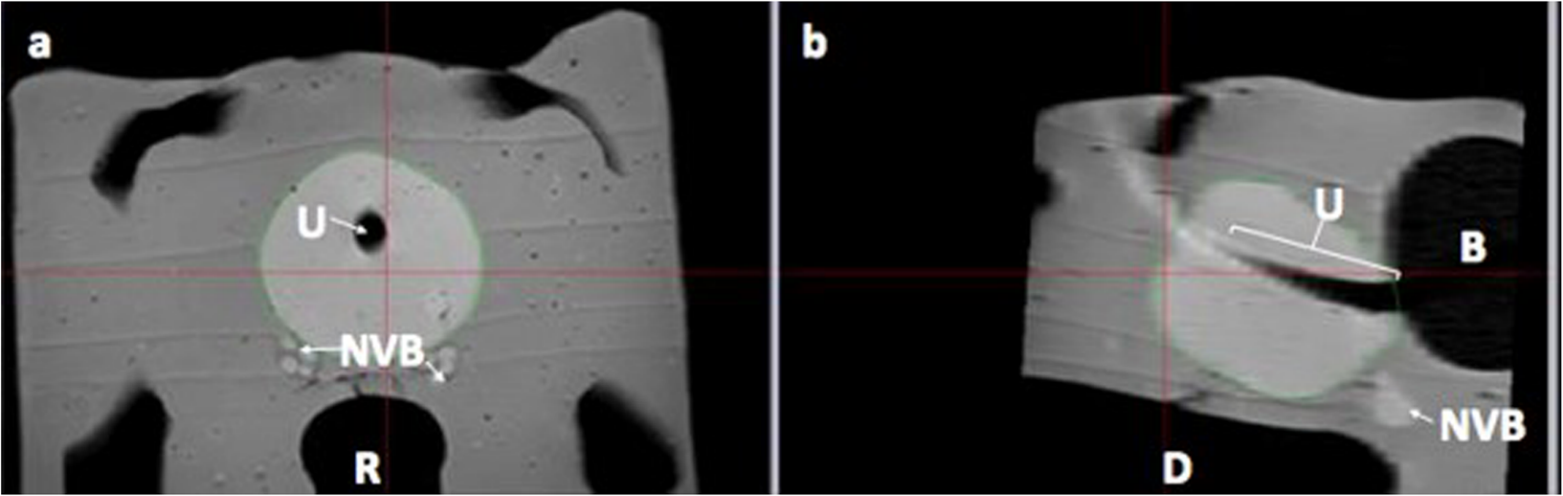
Contoured prostate capsule. **a** Axial and (**b**) sagittal T2-weighted magnetic resonance images. Note the pelvic phantom model urethra (U), rectum (R), bladder (B), and neurovascular bundles (NVB)

This study was performed on one custom pelvic anthropomorphic phantom model (Fig. 3), using the prototype TFC with six degrees of freedom electromagnetic tracking sensors that enable spatial localisation and custom National Institutes of Health fusion software OncoNav (Fig. 4) with an electromagnetic field generator (Uronav, InVivo, Philips Healthcare, Best, Netherlands). The phantom underwent 3-T MRI (Achieva, Philips Healthcare, Best, The Netherlands) with 32-channel cardiac coils, and the prostate capsule was segmented/contoured manually by the expert software engineer, with more than 10 years of experience in prostate capsule segmentation on MRI and US. The phantom prostate capsule margins were superimposed on axial and sagittal T2-weighted images and transferred to the custom FBx workstation for study trials (Fig. 5).

First, the TFC was placed in the phantom urethra and bladder, and the balloon was inflated with water to secure the TFC in place. Second, an US three-dimensional volume reconstruction of the model prostate was performed after a two-dimensional transrectal US transducer “sweep” (Prostate Triplane 8818, BK Ultrasound, Peabody, USA) from base to apex. Third, the TFC FBx user segmented the phantom prostate edge on triplane US images. Fourth, the MRI volume was registered and fused to the US volume using the custom fusion software. This process was repeated for each trial.

This study consisted of two parts: part A and part B. During part A, the data were collected from intra-experimental screen captures after custom software registration and fusion of MRI/US data, both with and without TFC input. During part B, a copper wire (needle) was placed as closely to prescribed targets as possible, under fusion guidance. The motion induced during part A was both rotational and translational, while only translational motion was induced during part B. Translational motion was created by dropping the computed tomography table, and rotational motion was created by lowering one side of the phantom. Six fiducial marker locations (placed on phantom surface, visible on computed tomography scans) were documented before and after combined motion. Fiducial markers were displaced by the “motion” an average of 12.11 mm with translation and 8° from the horizontal with rotational motion.

**Fig. 6:**
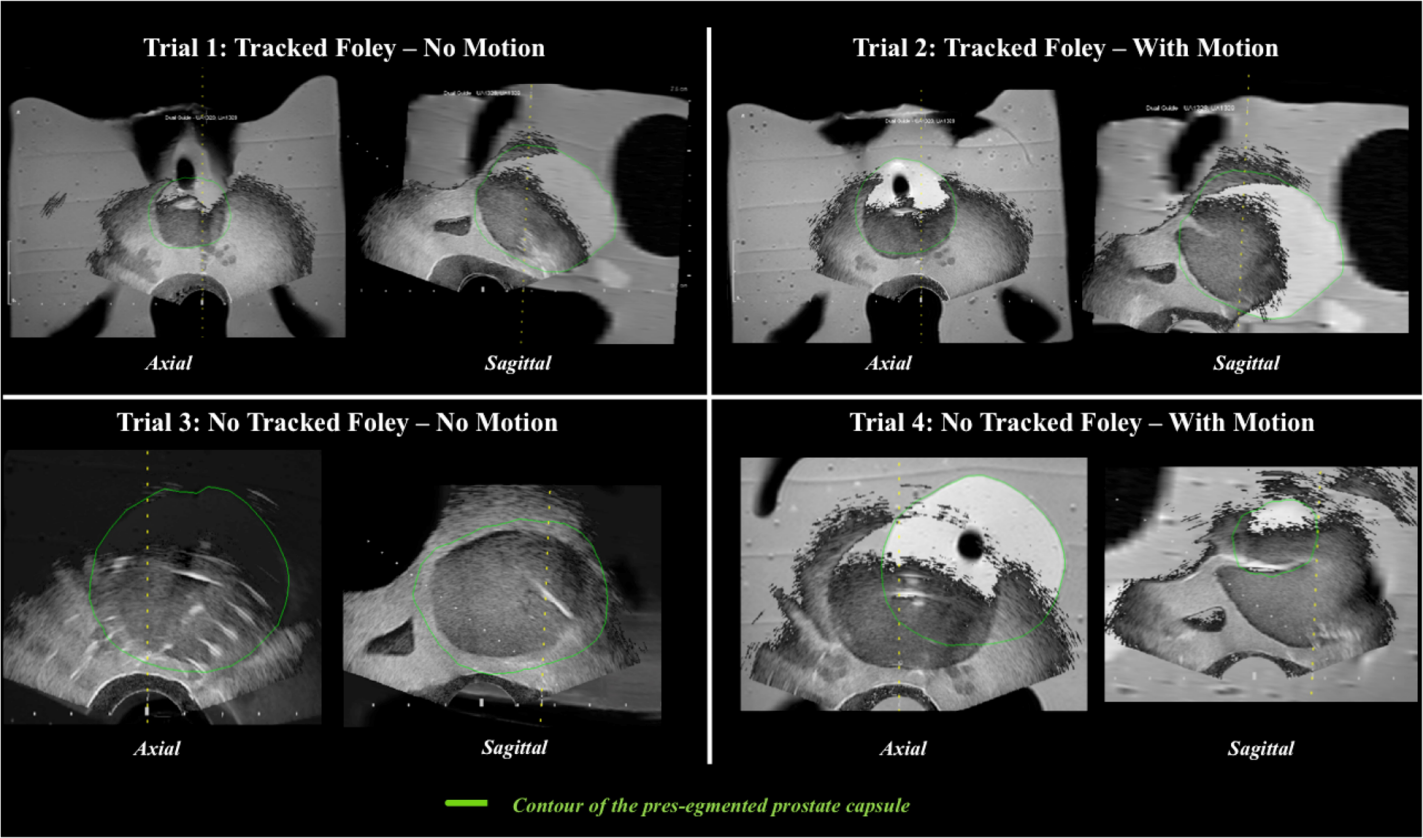
Intra-experimental screen captures from trials 1 to 4: magnetic resonance imaging (MRI)/ultrasound (US) fusion images, displayed with variable windowing blending MRI and US. Note trial 3 images showing the US predominantly on the left and more MRI on the right. The green contour is the pre-segmented prostate capsule from the pre-procedural MRI

**Fig. 7:**
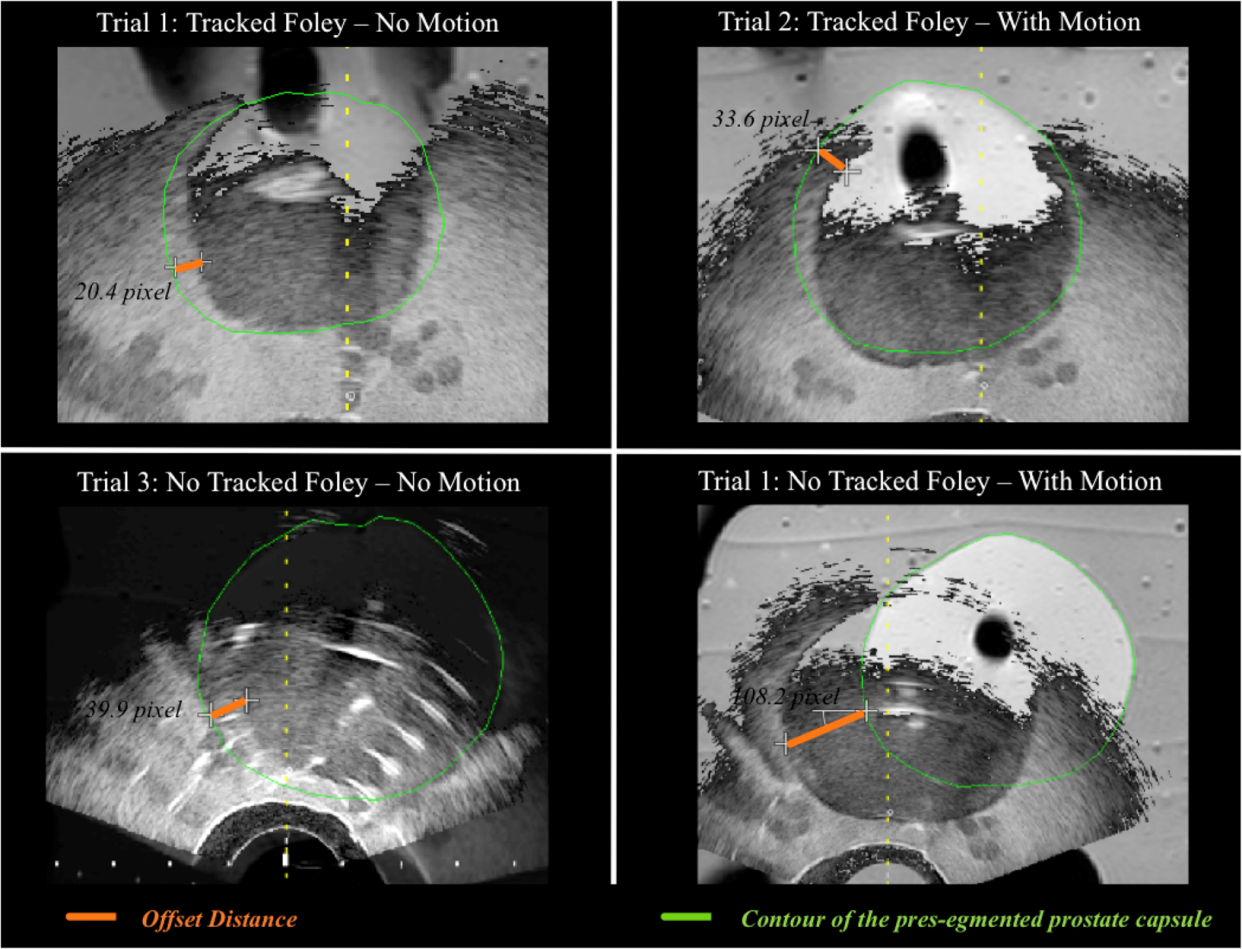
Maximum offset distance (OD) between the phantom edge on ultrasound (US) and magnetic resonance imaging (MRI) on the axial plane. OD was defined as the distance between the phantom edge on US and perpendicular line striking the same location as seen on the contoured edge on MRI. The green contour is the pre-segmented prostate capsule from the pre-procedural MRI

During part A, four trial scenarios were created which varied the artificial motion and the presence and absence of TFC input. Screen captures were collected after fusion of MRI/US images during each trial: (1) TFC no-motion, (2) TFC with motion, (3) no-TFC no-motion, and (4) no-TFC with motion. Trials involving no motion were performed first, followed by trials involving motion. Next, Dice similarity coefficients (DSCs) were calculated from screen captures viewed on a picture archiving and communication system in which the prostate edge was best seen on both US and MRI (two per trial: one axial and one sagittal view). Average maximum offset distances (ODs) were calculated from all screen captures (Fig. 6) across the four scenarios. OD was defined as the distance between the phantom edge on US and perpendicular line striking the same location as seen on the contoured edge on MRI (Fig. 7).

Part B began with the phantom positioned neutrally, repeating the US sweep to build the three-dimensional volume, volume reconstruction, prostate edge segmentation on US, and finally MRI/US fusion using the custom fusion software. Three targets were identified (beginning and end of the neurovascular bundles seen on CT and US), overlaid with a virtual marker that was then targeted with a needle during the same four trials scenarios (trials 1 to 4, above). Artificial motion was induced by dropping the computed tomography table 15 mm for trials 2 and 4. For part B, only translational motion was induced, without rotational motion. After a needle was placed in each target location, assisted by the custom fusion software, system error (SE) was calculated from computed tomography (Brilliance 16, Achieva, Philips Healthcare, Best, The Netherlands). SE was defined as the distance from the tip of the needle to the location of the intended target (neurovascular bundles).

Data were checked for normal or near-normal *versus* non-normal distribution using the Shapiro-Wilk test at 0.05 significance and reported as mean ± standard deviation. ODs were compared using a *t* test at a significance level of 0.05. Prostate contour two-dimensional area measurements were made on screenshots on MRI, US, and then the area of MRI; US overlap in pixels was used to calculate DSC for each trial. SE distances were compared using a *t* test at a significance level of 0.05. StataSE 15 software was used (StataCorp, College Station, USA).

## Results

**Fig. 8:**
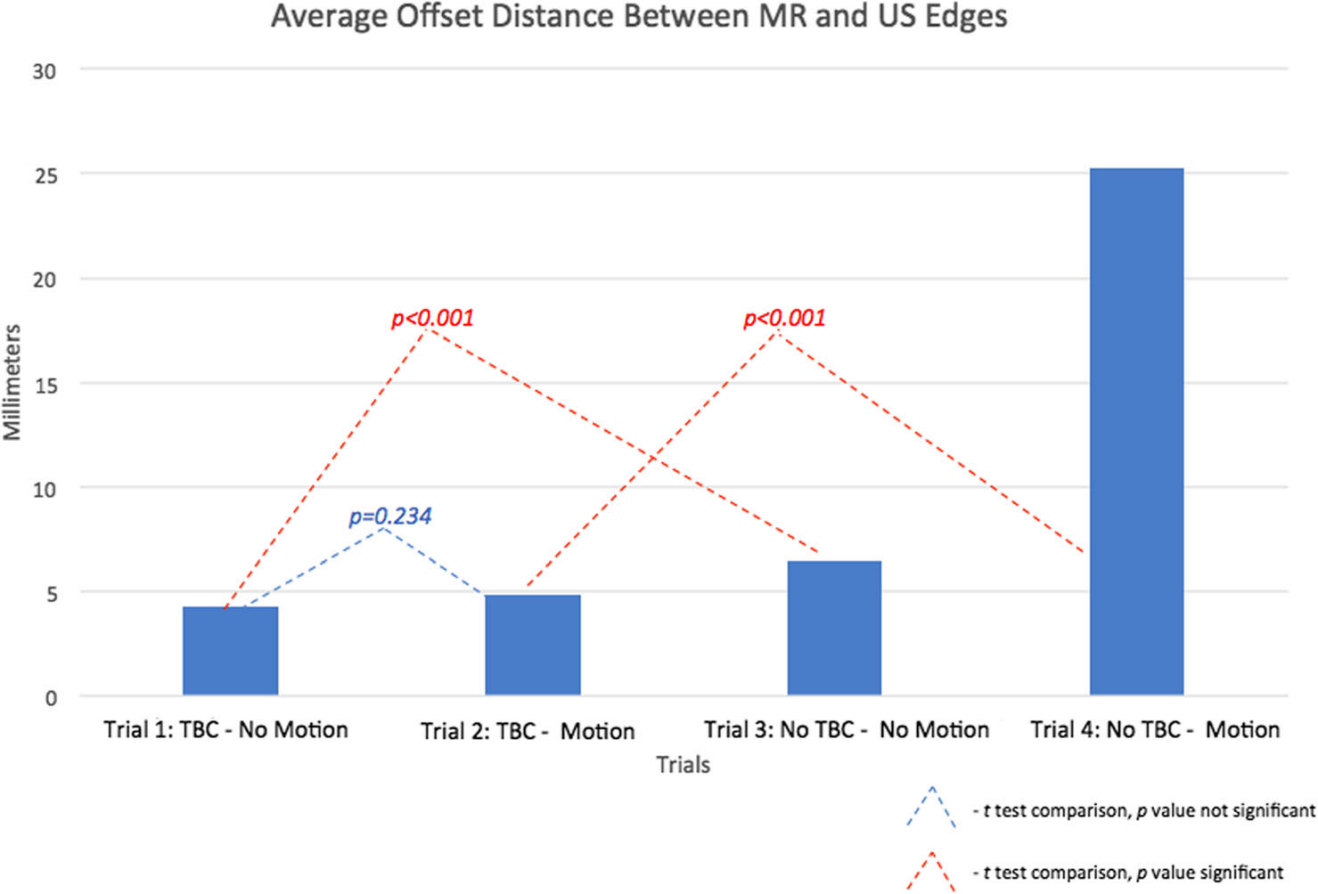
Average offset distance (OD) and comparison of results for trials 1 to 4. OD was defined as the distance between the phantom edge on ultrasound and perpendicular line striking the same location as seen on the contoured edge on magnetic resonance imaging on fusion images. Trials vary the presence/absence of the TFC and motion (see text)

During part A, 40 total screen captures (*n* = 80 total OD measurements) were saved to compare the 4 trials (10 for each of the 4 trials). OD was significantly lower when the TFC was present *versus* absent both with motion (trial 2 *versus* trial 4, 4.85 ± 1.60 *versus* 25.29 ± 6.50 mm, *p* < 0.001) and without motion (trial 1 *versus* trial 3, 4.29 ± 1.24 *versus* 6.42 ± 2.29 mm, *p* < 0.001). There was no significant difference in OD before and after motion when the TFC data was present (trial 1 *versus* trial 2, 4.29 ± 1.24 *versus* 4.85 ± 1.60 mm, *p* = 0.234). When the TFC was absent, there was significantly higher OD after artificial motion (trial 3 *versus* trial 4, 6.42 ± 2.29 *versus* 25.29 ± 6.50 mm, *p* < 0.001). The mean DSC for trials 1, 2, 3, and 4 was 0.88, 0.88, 0.74, and 0.61, respectively, for the 8 screen captures in part A. Results concerning the study part A are summarised in Fig. 8.

**Fig. 9:**
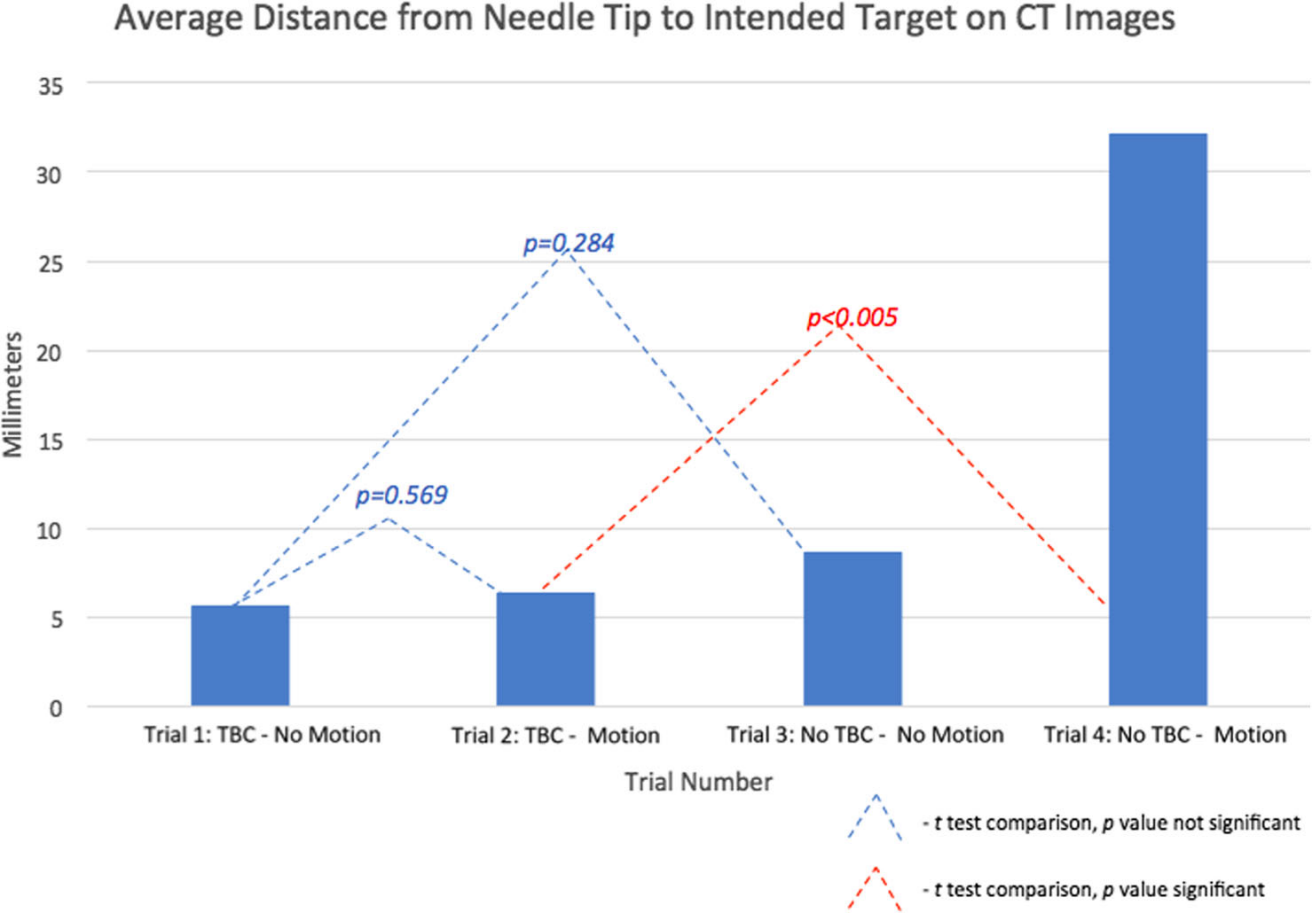
Average system error and comparisons for trials 1 to 4. System error was defined as the distance from the tip of the needle, placed under fusion image guidance, to the location of the intended target. Trials vary the presence/absence of the TFC and motion (see text)

During part B, 12 computed tomography scans were performed (*n* = 12 total SE measurements) to compare between the 4 trials. SE was significantly smaller when the TFC was present *versus* absent with motion (trial 2 *versus* trial 4, 6.35 ± 1.31 *versus* 32.16 ± 6.50 mm, *p* < 0.005). There was no significant difference in SE before and after motion when the TFC was present (trial 1 *versus* trial 2; 5.57 ± 1.50 *versus* 6.35 ± 1.31 mm, *p* = 0.569). When the TFC was absent, there was significantly higher SE after artificial motion (trial 3 *versus* trial 4, 8.63 ± 4.03 *versus* 32.16 ± 6.50 mm, *p* < 0.010). There was no significant difference in SE when the TFC was present *versus* absent and remained static (trial 1 *versus* trial 3, 5.57 ± 1.50 *versus* 8.63 ± 4.03 mm, *p* = 0.284). Results concerning the study part B are summarised in Fig. 9.

## Discussion

This study demonstrated that the TFC can provide active intra-procedural fusion image correction automatically after simulated motion in a model pelvis. Inaccuracies in targeting pre-identified prostate locations are often multi-factorial, and may be related to prostate motion or deformation, patient movement, or imaging challenges, including impaired segmentation, image registration or mismatch, and offset of imaging planes [7, 25]. The TRUS plane for acquisition, registration, and manual intra-procedural alignment correction is slightly offset from that of the MRI, adding to the challenge [26]. Current FBx platforms and software systems inadequately compensate for motion of the prostate, thus limiting the accuracy of this technology, even when the prostate is mostly stationary and static. Additionally, registration methods and techniques are widely variable and non-standardised across many commercial platforms, contributing toward variable outcomes from those centres performing or reporting image-guided prostate procedures [27]. Standardisation of rigid and elastic registration combined with dynamic referencing of the prostate might reduce the operator variability and the inaccuracies induced by subjectivity, experience, and variable intra-procedural corrections of misaligned fusion images.

Within the TFC, six degrees of freedom tracking sensors induce a weak current in the presence of a rapidly changing magnetic field, enabling spatial localisation on imaging (same principle of electromagnetic tracking in existing FBx systems) [18, 28]. The TFC resides within the urethra, a non-linear central pathway. This urethral path is less altered by prostate motion (especially rotational) or deformation, than other areas of the prostate, and can act as a standardisation tool or “home reference” for correcting fusion image mismatch during a procedure or surgery. Although speculative, it is possible that FBx can perhaps then be more accurately performed in the presence of prostate motion, since the motion of the urethra (and TFC within) can provide real-time referencing information, even with poor image visualisation. The fusion image mismatch can thus automatically be accounted for, and a correction may be computed and applied to restore the initial registration in real time.

During image-guided prostate procedures, the patient often moves involuntarily due to pain, discomfort, and/or pressure related to the insertion of needles or TRUS transducer. Moreover, a TRUS transducer can cause distortion of the contour of the prostate during the FBx. Finally, focal blood, local anaesthetic, bowel gas, dissection, bladder filling, or respiratory motion of the patient may cause shifting of the prostate, especially when the patient is in the prone position [18]. The TFC has the ability to maintain appropriate MRI/US fusion image alignment after simulated organ/patient motion in a catheterisable pelvic phantom model. All three methods of evaluation (DSC, OD, SE) supported the potential benefit of the TFC.

The TFC core concept of active intra-procedural fusion image correction may potentially be applied to other procedures, settings, or organs with additional hardware components and software [18]. The TFC ability to maintain accurate image alignment after motion could not only benefit existing procedures using fusion imaging (FBx, trans-perineal FBx, focal PCa ablations), but also potentially enable fusion guidance within robotic platforms [8, 12, 15, 16, 19–24, 29–31]. In addition to aid in alignment after simulated prostate motion, this study also indicated that the TFC may aid in more accurate image alignment while the patient remains static. It is possible this finding may stem from the fact that there is some degree of organ motion or deformation when the US transducer is introduced in the rectum, either during the initial US sweep or at any other point during image-guided procedures. The adjustments provided by the TFC may automatically correct for this subtle organ motion or deformation. Our data indicated increased fusion image alignment when the TFC correction was available and no simulated motion was induced, reporting significantly smaller OD in this scenario. Reported DSC scores showed a similar trend. Although the TFC SE was favoured when compared to no TFC SE when static, the results were not statistically significant.

Standardisation and reproducibility of current image-guided prostate procedures have been less than ideal [5, 9, 10]. Accordingly, there is an urgent need for hardware, systems, and methods for maintaining accurate registration between pre-procedural and intra-procedural images. Image-guided prostate procedures require operator experience for reliable registration of mpMRI/US images and for accurate targeting of pre-identified areas [8, 17, 32]. The TFC may help with the issue of prostate motion by providing a standardised/semi-automated method of maintaining the original registration. Moreover, computer-assisted biopsy systems may facilitate more widespread and standardised use of FBx, even by novice operators who may be uncomfortable manipulating dedicated navigation software [25, 32]. On the other hand, some intrinsic limitations are still present such as the general contraindications for MRI (*e.g.,* the claustrophobia and the presence of a magnetic field or of the pacemakers) and the current failure rate of TRUS-guided biopsy [12, 33–36]. Additional research may explore the extent to which the TFC aids in fusion image alignment when the prostate is static, specifically among novice FBx users.

In our study, fusion image alignment with and without the TFC was compared in three ways with similar results and one method physically targeted region of interests with needles. However, there were several limitations of this study. The physical targeting of identified structures with needles damaged the phantom model somewhat and limited the number of SEs that could be measured on one custom anthropomorphic phantom. There was also no easy and effective way to blind the operator to the presence or absence of the TFC and artificial motion without crippling the study. It remains to be seen whether (and in which scenarios) this solution might prove worthy of the cost and risks associated with an additional catheterisation procedure. Also speculative is the eventual utility for addressing intra-operative prostate organ motion during robotic prostatectomy, which suffers even greater dynamic referencing challenges and shortcomings than FBx. The TFC merits evaluation in a clinical trial setting for biopsy, ablation, and robotic prostatectomy.

In conclusion, fusion-guided prostate procedures have proven benefits. However, fusion biopsy is not yet standardised and suffers from variability and challenges in maintaining accurate image alignment as well as a dearth of tools for facilitated and semi-automated registration. Challenges remain in maintaining registration integrity, procedural standardisation, and reproducibility that may limit the benefits or adoption of fusion guidance. A custom TFC maintained accurate fusion image alignment after movement and provided significantly better fusion image alignment when static. The inherent challenges of maintaining fusion image alignment may be addressed by this additional smart device, in the presence of an electromagnetic field generator. Although yet to be proven, a TFC may help address clinical needs related to standardisation and correction of intra-procedural prostate motion during fusion-guided prostate procedures such as biopsy, ablation, or robotic prostatectomy.

## Data Availability

The datasets used and/or analysed during the current study are available from the corresponding author on reasonable request.

## Abbreviations

DSC: Dice similarity coefficient
FBx: Fusion-guided biopsy
mpMRI: Multiparametric magnetic resonance imaging
MRI: Magnetic resonance imaging
OD: Offset distance
PCa: Prostate cancer
SE: System error
TFC: Tracked Foley catheter
TRUS: Transrectal ultrasound
US: Ultrasound
